# Deviation from Mendelian transmission of autosomal SNPs can be used to estimate germline mutations in humans exposed to ionizing radiation

**DOI:** 10.1371/journal.pone.0233941

**Published:** 2020-10-27

**Authors:** Hugo Pereira Leite Filho, Irene Plaza Pinto, Lorraynne Guimarães Oliveira, Emília Oliveira Alves Costa, Alex Silva da Cruz, Daniela de Melo e Silva, Claudio Carlos da Silva, Alexandre Rodrigues Caetano, Aparecido Divino da Cruz

**Affiliations:** 1 Programa de Pós-Graduação em Biotecnologia e Biodiversidade, Universidade Federal de Goiás, Goiânia, Goiás, Brazil; 2 Universidade Estadual de Goiás, Anápolis, Goiás, Brazil; 3 Núcleo de Pesquisa Replicon, Mestrado em Genética, Escola de Ciências Agrárias e Biológicas, Pontíficia Universidade Católica de Goiás, Goiânia, Goiás, Brazil; 4 Programa de Pós-Graduação em Genética e Biologia Molecular, Universidade Federal de Goiás, Goiânia, Goiás, Brazil; 5 Laboratório de Genética Molecular e Citogenética Humana, Laboratório Estadual de Saúde Pública Dr. Giovanni Cysneiros, Secretaria de Saúde Pública do Estado de Goiás, Goiânia, Goiás, Brazil; 6 Embrapa Recursos Genéticos e Biotecnologia, Brasília, Distrito Federal, Brazil; ENEA Centro Ricerche Casaccia, ITALY

## Abstract

We aimed to estimate the rate of germline mutations in the offspring of individuals accidentally exposed to Cesium-137 ionizing radiation. The study included two distinct groups: one of cases, consisting of males and females accidentally exposed to low doses of ionizing radiation of Cs^137^, and a control group of non-exposed participants. The cases included 37 people representing 11 families and 15 children conceived after the accident. Exposed families incurred radiation absorbed doses in the range of 0.2 to 0.5 Gray. The control group included 15 families and 15 children also conceived after 1987 in Goiânia with no history of radiation exposure. DNA samples from peripheral blood were analyzed with the Affymetrix GeneChip^®^ CytoScanHD^™^ to estimate point mutations in autosomal SNPs. A set of scripts previously developed was used to detect *de novo* mutations by comparing parent and offspring genotypes at the level of each SNP marker. Overall numbers of observed Mendelian deviations were statistically significant between the exposed and control groups. Our retrospective transgenerational DNA analysis showed a 44.0% increase in the burden of SNP mutations in the offspring of cases when compared to controls, based on the average of MF_MD_ for the two groups. Parent-of-origin and type of nucleotide substitution were also inferred. This proved useful in a retrospective estimation of the rate of *de novo* germline mutations in a human population accidentally exposed to low doses of radiation from Cesium-137. Our results suggested that observed burden of germline mutations identified in offspring was a potentially useful biomarker of effect to estimate parental exposure to low doses of IR and could become an important marker suitable for biomonitoring human population exposed to environmental mutagens.

## Introduction

In 1987, a series of unexpected events resulted in a major radiological accident in Goiânia, Goiás, Brazil, causing human, animal, plant and environmental exposure to gamma ray ionizing radiation (IR) of Cesium-137 and contamination by the radionuclide [1]. For some people, individual exposure resulted from internal and external contamination by the radioactive salt, while others were exposed to radiation emitted by the decay of Cesium-137. In some cases, people were both exposed to radiation and contaminated by the radionuclide. In the aftermath, 249 people were exposed to IR from Cesium-137, leading to individual absorbed doses of IR ranging from 0 to 7 Gy, resulting in four fatalities during the acute phase of the accident [2, 3].

Following the accident, the exposed population has been extensively monitored using genetic biomarkers, as they have been shown to be efficient biomarkers of exposure to gamma rays [4]. However, each biomarker tends to reveal a distinct biological phenomenon in the exposed cells, mostly associated with DNA repair and how cells physiologically coped to survive a specific insult. In this context, our group and others have established somatic mutation frequencies using data from glycophorin A [5] and HPRT assays [6], chromosomal aberrations [7–9], BCL2/J(H) translocation [10], and micronucleus frequencies [11] in T-lymphocytes of the cohort accidently exposed to Cesium-137 IR. Moreover, in order to understand the effect of IR on the induction of germ line mutations, STR markers [12] were initially used to estimate the rate of germline mutations in the offspring of the exposed cohort. More recently, CNVs have been used as biomarkers for parental exposure to demonstrate the effect of low absorbed doses of IR on germline mutations in the cohort’s offspring conceived after the accident [13].

Radiation-absorbed dose relates to the estimated quantity of energy deposited in the mater per unit of mass. Thus, it can be used as an indirect measurement of the harmful biological effect of the radioactive energy on the cellular system. It is calculated by estimating the concentration of energy from radiation exposure deposited in each organ, using a reference value, the type of radiation and the potential for radiation-related mutagenic changes in each organ or tissue [14].

The exposure of cells to IR delays the normal progression of the cell cycle [15–17], initially observed as a passive cellular response resulting from of the induction of DNA damage in the exposed cells. The irradiated cell must adapt to the insult and facilitate DNA repair processes, especially fixing double-strand breaks, the most common damage after DNA exposure to IR [18–22].

The mutagenic effects of IR on the human germ line cells are of concern, as they lead to the accumulation of mutations in the offspring of irradiated parents, amounting to an increase in the mutational burden [23]. Despite numerous studies, little is known about the genetic effects of low doses of radiation from low linear energy transfer gamma radiation exposure in humans. Most of the consolidated evidence comes from the extrapolation of the induction of germline mutations in mammals, often rat and mouse models [24, 25].

Advances in the methodologies of genomic analysis have greatly increased the volume of nucleotide sequence data, enabling the identification of thousands of SNPs (single-nucleotide polymorphisms). Variations in SNPs are important to determine genotypic and phenotypic relationships, within and between species and populations, and also to identify variants related to genetic diseases in humans and animals [26]. In this context, genomic analysis can be a useful tool to study and understand the effects of IR exposure on animals and humans [13, 27].

In recent decades, several genotyping technologies have been developed to characterize SNPs all producing genotype matrices with hundreds of thousands of datapoints. Algorithms based on parametric and nonparametric statistical models have been used to determine the genotype of each SNP from the fluorescence signal intensity of marked probes, which are scanned, captured, and arranged in a matrix format [28, 29]. One commercially available SNP array, the GeneChip^®^ CytoScanHD^™^ (Thermo Fisher Scientific, Waltham, MA, USA), is considered to be a high-density matrix, including about 750,000 polymorphic markers with an average genotyping accuracy of >99% [30].

In the aforementioned context, the general objective of the current study was to quantify Mendelian deviations (MD) in genome-wide autosomal SNP data from a cohort of people conceived after parental exposure to Cesium-137 IR, and a group of non-exposed people from the same geographical area. The rate of MD was applied to evaluate if the observed burden of germline mutations identified in the offspring could be a potentially useful biomarker of parental exposure to low doses of IR.

## Material and methods

### Sample collection, processing, and genotyping

The experiment was designed as a case-control observational study. The group of cases consisted of 11 families, of whom at least one of the parents was accidentally exposed to IR during the Cesiu-137 accident, totaling 37 participants (11 fathers, 11 mothers, and 15 children conceived after the accident). The radiation absorbed doses for the exposed parents ranged from 0.2 to 0.5 Gy [3, 13]. As controls, biological samples were obtained from 15 families living in Goiânia since the time of the accident with no prior history of exposure to IR. Thus, the control group was comprised of 15 fathers, 15 mothers, and 15 children also conceived after 1987. A total of 82 subjects were used in the study whose DNA samples were analyzed using the SNP-array GeneChip^®^ CytoScanHD^™^ (Thermo Fisher Scientific).

Cases and controls participated voluntarily in the study, which was approved by the ethics committee on research with humans from the Pontifical Catholic University of Goiás (PUC-Goiás)–CAAE number 49338615.2.0000.0037. At the time of blood collection, participants answered a lifestyle questionnaire and signed an informed consent form. A total of 10 mL of peripheral blood in EDTA was voluntarily donated by all participants. Total genomic DNA was isolated from whole blood using Illustra blood genomicPrep Mini Spin Kit® (GE Healthcare, Milwaukee, WI, USA) and stored at −20°C. The remaining biological material was stored according to CNS Resolution 441/11.

Chromosomal microarray analyses were carried out in GeneChip CytoScanHD^®^ arrays (Thermo Fisher Scientific) in order to collect individual genotypes from polymorphic autosomal markers. SNP genotypes were generated using ChAS^©^ (Thermo Fisher Scientific). Every array met the quality controls recommended in the manufacturer’s guidelines. SNP genotypes were filtered based on individual call confidence levels for each marker, thus calls with confidence levels <5x10^-2^ and invalid (no call or null) in one or more samples were removed from the dataset. Therefore, only markers with quality-controlled genotypes in all samples were considered for the analysis. Genotyping was based on the hg19 version of the human genome hosted on the UCSC Genome Browser (University of California, Santa Clara, CA, USA). We also applied the CpG island track from UCS browser in order to stablish the rule out C>T mutations at CpG sites. As the array genotypes didn’t allow the discrimination from which strand the damage was derived, all substitutions were included in the data sets.

### Principle component analysis

Principle Component Analysis (PCA) methods were used to assess whether participants in the case and control groups came from the same genetic population, the dataset contained about 522K SNPs. This step was also included to assess whether individual sample quality effects may have generated spurious results. SNPs with minor allele frequencies (MAF) below 0.01 were removed from the dataset, including all mendelian errors in the samples. Data pruning of the final dataset was performed using the PLINK (2.0) package [31] to generate a subset of markers for PCA analysis using the following parameters: window size of 500 SNP with a step size of 5 SNP, using an r^2^ threshold of 0.1. Pruning resulted in a subset of 2.789 SNPs that were used to estimate principal components and to generate plots for each test group.

### Analysis and phasing of genotyping data to identify mendelian deviations

**Fig 1:**
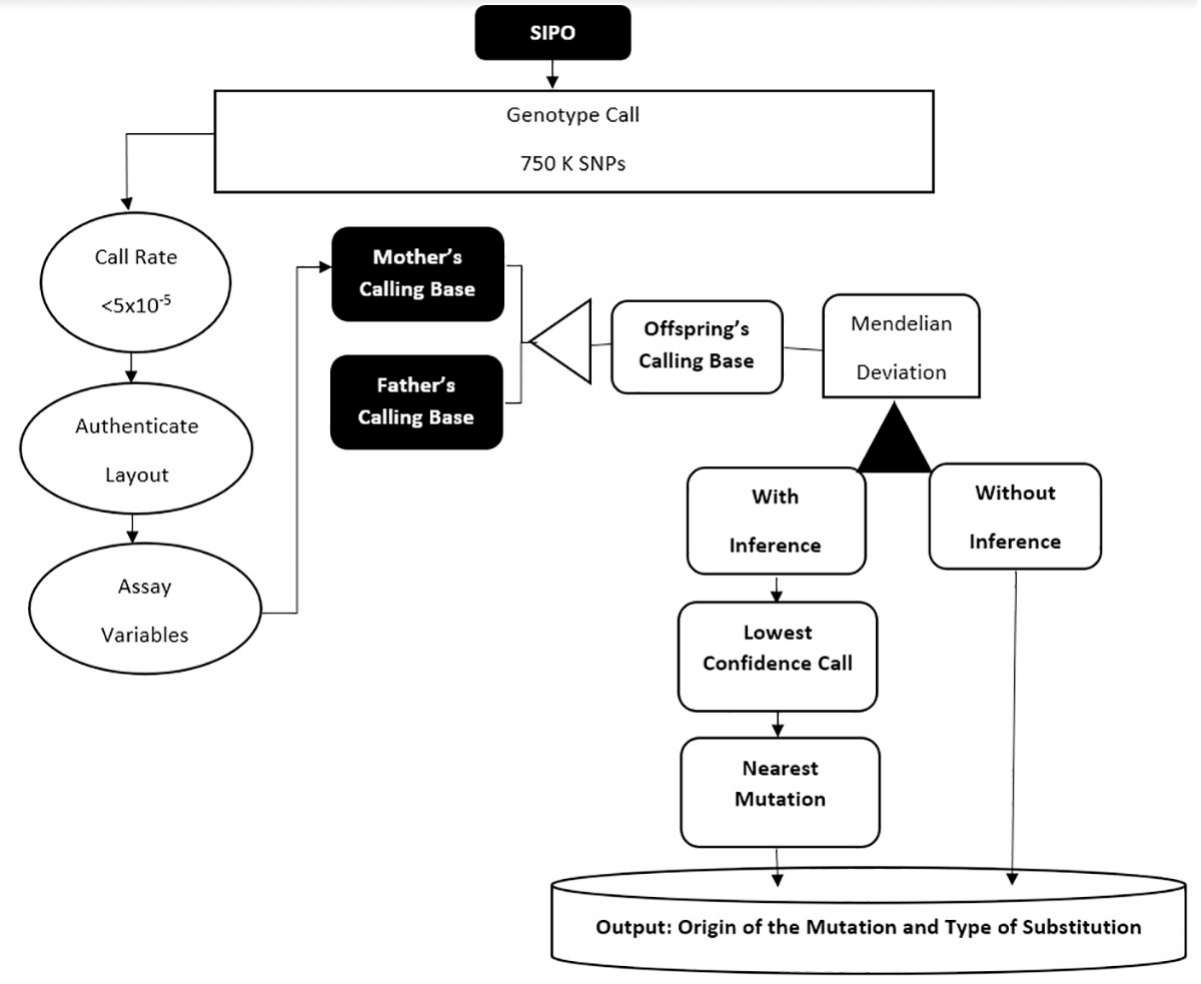
Steps performed by SIPO to infer de novo mutations. Deducting the parental origin of the MD, indicating the type of substitution and generating the estimated rate of Mendelian deviation in the offspring of people accidentally exposed to Cs-137 ionizing radiation.

MDs were inferred with a set of previously developed Perl scripts and R libraries [32] termed SIPO (Scripts for Inference of Parental Origin) to mine SNP data in MySql^©^ format. The SIPO pipeline was listed in (Fig 1) and supporting information files are accessible in a GitHub under the accession URL: https://github.com/hugofilho/sipo. Parent genotypes were compared with respective offspring genotypes for each individual marker. Sex chromosome data were excluded from the analysis, as X-linked data showed elevated noise and Y-specific regions had low marker coverage. Table 1 shows all data variables considered by SIPO.

**Table 1:** Variables generated by ChAS^®^ and considered by SIPO to identify MDs.

Marker	SNP marker identifier
**Genotyping**	Genotyping call. Biallelic information. Three possible combinations: AA/BB/AB
**Trust Value**	Confidence value for each genotyping call
**Sign A**	Gross sign value for sign A on the marker
**Sign B**	Gross sign value for sign B on the marker
**Nitrogen Base**	Call from the nitrogen base. Biallelic information. Ten possible combinations: AA|AC|AG|AT|CC|CG|CT|GG|TT
**dbSNP**	SNP identifier record in the NCBI dbSNP database
**Chromosome**	Autosome associated with the marker
**Chromosome Position**	SNP locus on the chromosome

**Fig 2:**
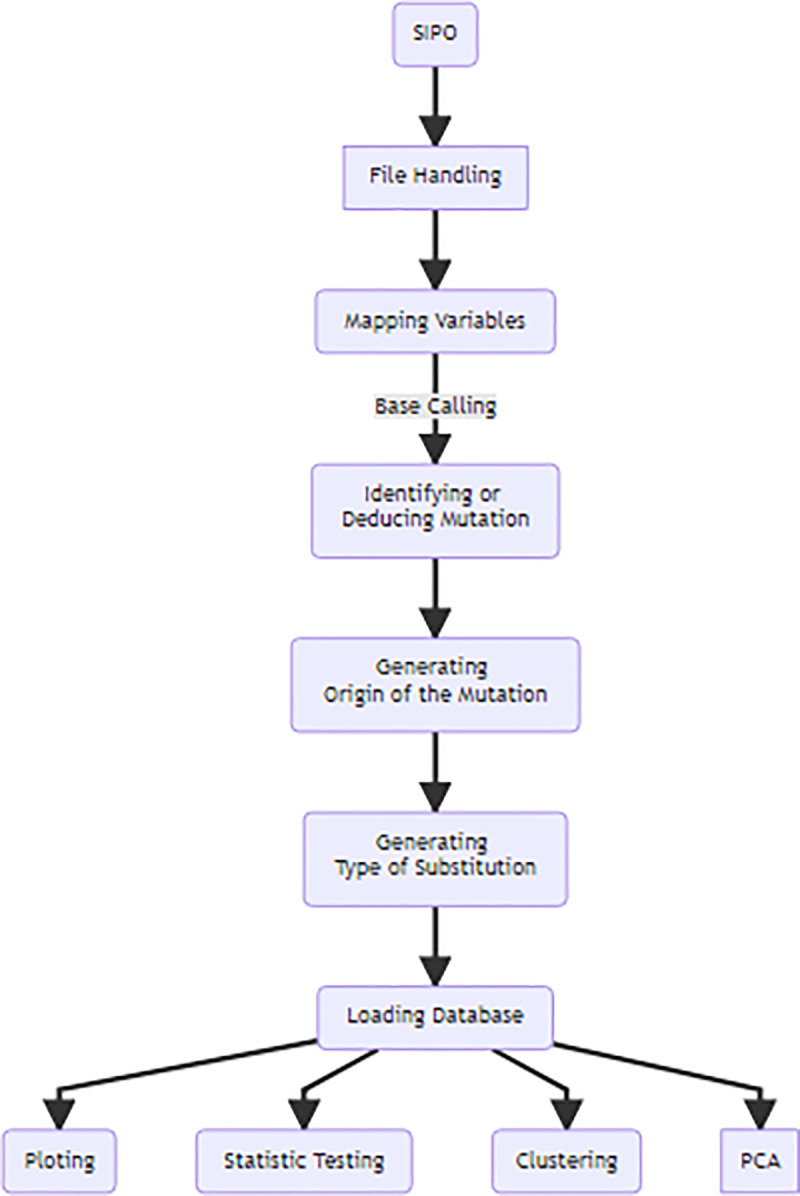
Workflow of the SIPO. Steps performed to infer de novo mutations from SNPs obtained from a high-density genotyping array.

First, SIPO validated the.CYCHP file generated by ChAS^®^, then SIPO identified trio variables and started to generate inferences for *de novo* mutations, corresponding to MDs in the child. Parental origin of observed mendelian deviations were inferred using basic expected mendelian inheritance rules applied over family trio data. For instance, if parent 1 had a genotype "AA" and parent 2 had a genotype "CC", and their child had genotype "GC", the germline mutation was inferred to parent 1. Executed steps allowed to determine nucleotide substitution type in addition to inferring the parent of origin of the MD observed in the offspring. Derived information was loaded into a MySQL database and R scripts were used to perform linear regression, clustering and PCA with the resulting data (Fig 2).

In some situations, SIPO was not able to identify the parental origin of a SNP based on Mendelian transmissions. To solve this challenge, two deductions were incorporated into the pipeline. The first deduction was coded into SIPO to identify confidence interval values of individual SNPs using ChAS^®^ data from the parents of a family trio. The second consisted of identifying the nearest mutated SNP, based on Euclidean distance using tools from Microsoft Excel^®^ (version 365), which had the parent of origin previously inferred following mendelian transmissions rules. Thus, at the end of the pipeline, the deductions aided to attribute the origin of a mutation to the parent who had both the lowest confidence interval value for that particular mutated SNP and who transmitted that chromosomal segment to the child based on the nearest variant SNP.

The total count of MD was used to estimate the germline mutation frequency (MF_MD_) in the offspring, using Eq 1: [13, 33]

$${MF}_{MD}=\frac{\sum T_{MD}}{b\;x\;nvp}$$ (1)

Where ΣT_MD_ = Total MD; b is a biallelic locus (2); *nvp* is the number of valid SNPs in the array according to the assembly of the human reference sequence (GRCh37/hg19) as indicated in S1 Table.

In the present study, all statistical tests were performed considering a 95% confidence interval and 5% significance level. The statistical tests used were the Shapiro-Wilk test, Student’s T test, regression analysis, clustering [34], and principal component analysis [35, 36]. The R statistical package [32] was used in all analyses.

## Results and discussion

The current study used SNP genotypes from a cohort of offspring born to parents accidentally exposed to Cs-137 to estimate the induction of germline mutations in humans exposed to low doses of ionizing radiation. As a cautionary note, in the current work, deviation of a Mendelian transmission implies that a point mutation observed in a child wasn’t observed in his/her parents, thus it was herein interpreted as a *de novo* mutation. However, we are aware that SNP variants can rise somatically due to DNA repair failure in the first cell divisions of the embryo, a variable common to both cases and controls and expected to be equally represented in the study datasets, bearing little bias to the dataset if any.

Before disclosing the results of the study, we also wish to note the limitation regarding the small size of the study cohort, which could render meaningful conclusions at first glance. In this context, two important rationales support the value of considering follow-up studies of human populations exposed to IR. First, considering the global effort with respect to radioprotection and regulation, it’s very unlikely that large accidentally exposed cohorts will be available world-wide to be investigated with the newest methodologies. Second, a high-density SNP array was used to call thousands of SNPs, covering a very large proportion of the genome. Thus, increasing the chances of identifying genomic variation in small populations that could be potentially useful to establish new biomarkers of effect to be applied in future studies investigating genotoxic and mutagenic responses to environmental stressors. The current available technologies applied to the study of genomes have that intrinsic characteristic, making them tools of first-tier choice in a variety of investigations, particularly when assessing small cohorts.

CytoScan HD Suite had an intrinsic algorithm, which allowed the analysis of a chromosome segment given the presence of polymorphic markers within that region. In the current study, the challenge was to establish the parent-of-origin for a point mutation based solely on Mendelian transmissions. In order to infer that origin, two deductions were incorporate into our pipeline, which allowed the inclusion of 9,522 and 4,821 MDs for case and control groups, respectively, into the dataset. In this context, the current pipeline could be used as an additional tool to define the parental origin of polymorphic variants obtained from SNP array genotypes.

**Fig 3:**
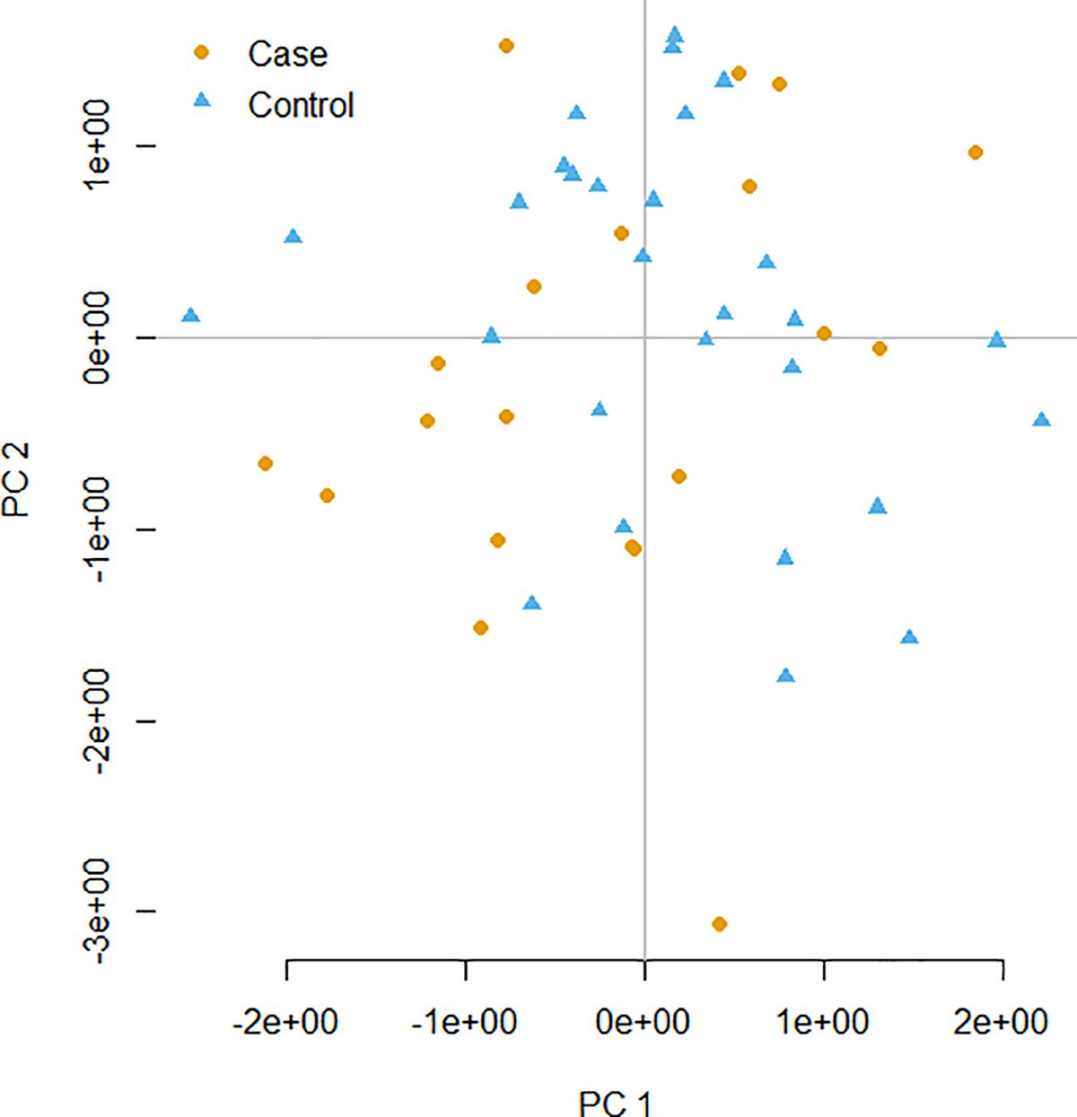
PCA with 2.7K SNP. The variables contained in the PCA represented the standardized relationship matrix by variance, multidimensional sizing (MDS) based on Hamming distances.

PCA results using a subset of LD-pruned data (522 Kb SNPs) indicated subjects included in both case and control groups belonged to the same population and there were no recognizable additional confounding factors associated with the test groups other than exposure to Cs-137 (Fig 3). Therefore, the MF_MD_ could be compared between groups, even with a reduced sample size. Observed MDs followed a normal distribution (*p = 0*.5592) and were all included in subsequent statistical analyses. The lowest individual numbers of MDs were 972 and 682, and the highest were 2,875 and 1,635 for the case and control groups, respectively (Table 2). Observed MDs were randomly distributed on the SNPs in the array. When performing family trio comparisons, most MDs (60%) were observed only once with no repetition, while 27%, 9% and 4% of the same MDs were respectively observed twice, three and four times in the family trios, confirming both the random effect of DNA damage induced by IR and spontaneous replication errors. Moreover, this observation also favors the quality of the array avoiding artefactual genotyping errors to be included in the dataset.

**Table 2:** Overall data from both control and exposed groups regarding the study of germline mutation in the offspring of people accidentally exposed to low absorbed doses of Cesium-137 ionizing radiation in Goiania (Brazil).

Group	Family	Exposed Progenitor	Absorbed Dose (Gy)	Paternal Age^2^^,^*	Maternal Age^2^	Age of offspring	Sex of offspring	MDs^1^				Total of valid SNPs	Frequency of MDs
								Father	Mather	Unknown	Total		
**Control**	Ct001	None	0	40	36	9	Female	783	694	10	1487	702,304	1,06E-03
	Ct25	None	0	47	36	9	Male	545	631	3	1179	683,381	8,62E-04
	Ct27	None	0	26	26	23	Male	594	729	9	1332	692,311	9,62E-04
	Ct39	None	0	24	24	3	Female	679	679	11	1369	674,320	1,02E-03
	Ct40	None	0	45	37	3	Male	710	711	30	1451	696,544	1,04E-03
	Ct45	None	0	37	31	15	Male	643	663	8	1314	683,243	9,62E-04
	Ct51	None	0	35	34	2	Female	384	479	8	871	697,737	6,24E-04
	Ct52	None	0	55	41	1	Male	1015	588	32	1635	710,477	1,15E-03
	Ct53	None	0	35	27	8	Male	315	361	6	682	713,372	4,78E-04
	Ct60	None	0	40	38	1	Female	501	482	6	989	712,261	6,94E-04
	Ct66	None	0	31	20	26	Female	543	594	2	1139	707,798	8,04E-04
	Ct68	None	0	33	20	10	Male	758	654	11	1423	712,191	1,00E-03
	Ct70	None	0	20	24	8	Female	448	545	3	996	714,892	6,96E-04
	Ct72	None	0	31	20	14	Female	521	614	4	1139	710,259	8,02E-04
	CtF09	None	0	19	21	25	Male	718	696	9	1423	712,352	9,98E-04
**Exposed**	Ex04	Father	0.1	27	27	20	Male	900	966	7	1873	698,305	1,34E-03
	Ex06	Father	0.3	35	26	9	Male	1045	1082	9	2136	693,858	1,54E-03
	Ex07-1F	Mother	0.2	54	24	19	Male	976	850	8	1834	692,375	1,32E-03
	Ex07-4F	Mother	0.2	56	26	17	Female	1509	1085	18	2612	689,467	1,89E-03
	Ex08	Mother	0.2	18	20	8	Male	538	718	4	1260	706,759	8,92E-04
	Ex10	Mother	0.2	21	24	2	Female	1187	1054	21	2262	705,993	1,60E-03
	Ex12	Mother	0.3	31	30	3	Male	1361	1486	28	2875	693,463	2,08E-03
	Ex15	Father	0.2	18	27	16	Male	560	645	7	1212	706,780	8,58E-04
	Ex18	Father	0.2	47	30	18	Male	819	800	12	1631	707,366	1,15E-03
	Ex21	Mother	0.2	38	27	20	Female	457	513	2	972	707,827	6,86E-04
	Ex22-2F	Mother	0.2	29	31	20	Female	1006	664	3	1673	707,075	1,18E-03
	Ex22-3F	Mother	0.2	32	34	17	Female	845	566	3	1414	708,278	9,98E-04
	Ex22-4F	Mother	0.2	33	35	16	Male	781	518	5	1304	708,885	9,20E-04
	Ex24	Father	0.5	21	19	12	Female	1010	1102	47	2159	703,635	1,53E-03
	Ex25	Father	0.5	18	16	15	Female	598	708	10	1316	706,598	9,32E-04

^1^Medelian deviations

^2^Age at conception

*All ages are in years old.

In the current study, mutation burden was defined by the number of *de novo* base substitutions in an assayed SNP of a child born to a parent exposed to IR. Thus, a total of 18,429 and 26,533 SNPs showed MD for control and cases, respectively. Thus, the overall frequencies of germline mutations observed in the different trios were, on average, 1.3 x10^-3^ and 0.9 x10^-3^ mutations per polymorphic marker. The Student’s T test showed the difference in the means was statistically significant assuming equal variances for both groups (p = 0.002). Tables 2 and 3 contain the summary of the data used in this study. Our retrospective transgenerational DNA analysis showed about a 44.0% increase in the burden of SNP mutations in the offspring of cases when compared to controls, based on the average of MF_MD_ for the two groups. The current study pioneered the application of SNP data analysis to identify MD and estimate germline mutations in the offspring of humans accidentally exposed to low absorbed doses of IR. Current findings corroborated our first study reporting the usefulness of small CNVs to estimate *de novo* human germline mutation rates in a similar cohort [13]. A previous study by [23] also described the usefulness of the mutation frequencies of *de novo* CNV and SNVs as biomarkers of effect for paternal exposure to IR in mice. Moreover, a recent study using whole genome sequencing data from an offspring of radar soldiers potentially exposed to IR found the differences in the frequency of *de novo* SNVs might be suited for the assessment of DNA damage from IR in humans [37].

**Table 3:** Summary of the descriptive data of the case and control groups for parental and F1 generations in the study of the effect of IR exposure on the induction of germline mutations in humans.

Generation	Variables		Cases	Control
**Parental**	N		15	15
	Age range (years)		16 − 56	19–55
	Mean age at conception (years ± SD*)	Paternal	31.9 (12.5)	34.4 (9.9)
		Maternal	26.4 (5.3)	28.9 (7.4)
	Absorbed dose (Gy)		0.2 − 0.5	0
**F1**	N		15	15
	Age range (years)		2 − 20	1–26
	Mean age (years ± SD*)		14.0 (5.9)	10.5 (8.5)
	Sex ratio (Male:Female)		8:7	8:7
	Mean MF_MD_ (± SD*)		1.3 x 10^−3^ (± 0.4 x 10^−3^)	0.9 x 10^−3^ (± 0.2 x 10^−3^)

*SD = Standard Deviation.

**Fig 4:**
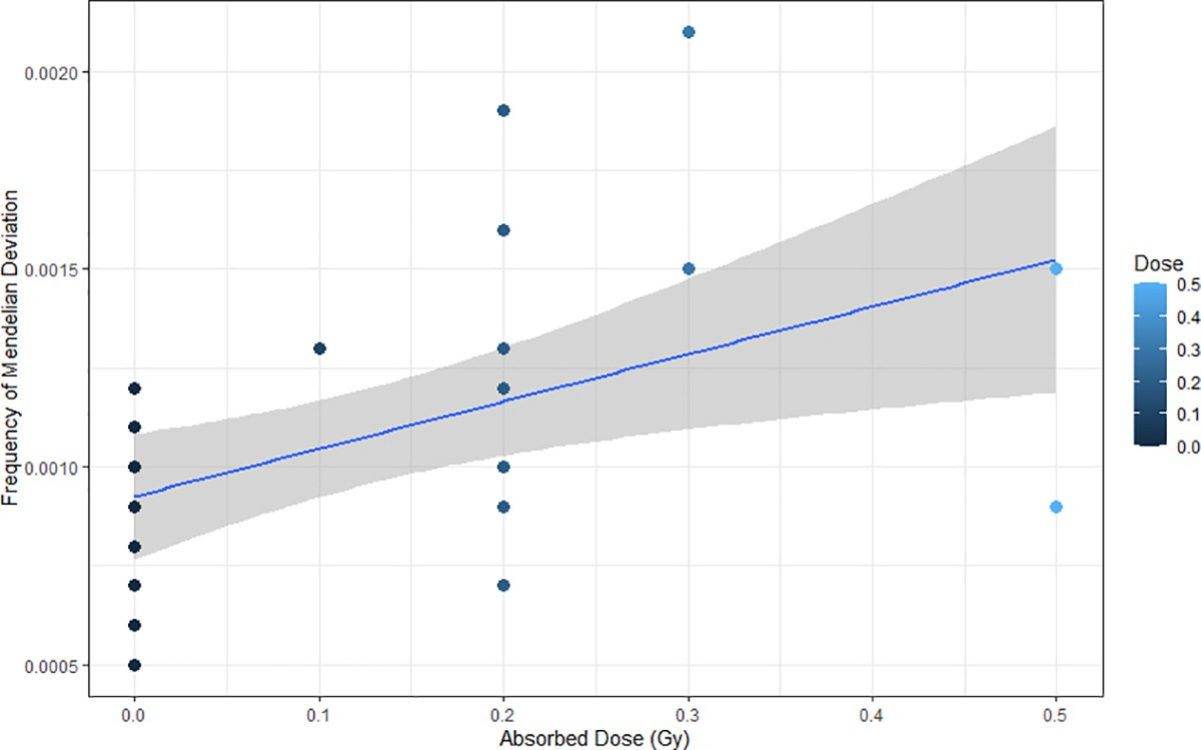
Representation the relationship between the radiation-absorbed doses and the means frequency of Mendelian deviations in a cohort of people conceived after parental exposure to ionizing radiation.

We also carried out a liner regression in order to evaluate the relationship between the radiation-absorbed doses and the MF_MD_ in our cohorts. Our results were statistically significant (p = 0.004; R^2^ = 0,257), suggesting that low absorbed doses of IR could predict an increase of the mendelian deviation in the exposed group, which could be linearly fitted (Fig 4) following the equation below:

$${MF}_{MD}=0.001+0.001(dose)$$

To date, there is extensive evidence supporting sex differences in mutation rates, with older fertile males expected to contribute more to the burden of a mutational health hazard than older females. A greater number of continuous cell divisions in the male germ line has been implicated as one reasonable explanation for such difference on paternal age effect [38, 39]. However, although this have been consistently reported, a clear and definite conclusion on the subject remains to be reached [38, 39]. In our study, the sex of the progenitors had no effect on the MF_MD_ of autosomal SNPs as for both case and control groups mothers and fathers contribute equal numbers of *de novo* MD to their offspring. When taken into consideration the sex of the exposed parent, the average of the frequencies of germline mutations of children born to exposed fathers was 1.2 x10^-3^ (±0.3 x10^-3^) and for exposed mothers was 1.3 x10^-3^ (±0.5 x10^-3^), with no statistical differences (p = 0.195) intragroup.

**Fig 5:**
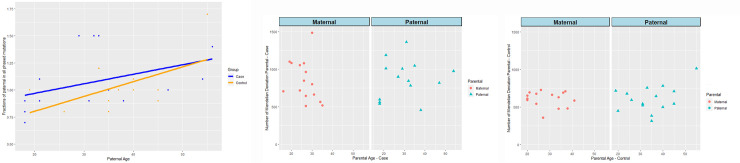
Potential parental age effect. (A) The fraction of paternal mutations as a function of paternal age at conception. Each point represents the data for one child (proband) with similar parental ages. The x-axis position is parental age, the y-axis position is fraction among paternal and maternal. Show the broadcast of data regarding the parental origin that transmitted the mutation to the child in the case group (B) and control group (C).

**Fig 6:**
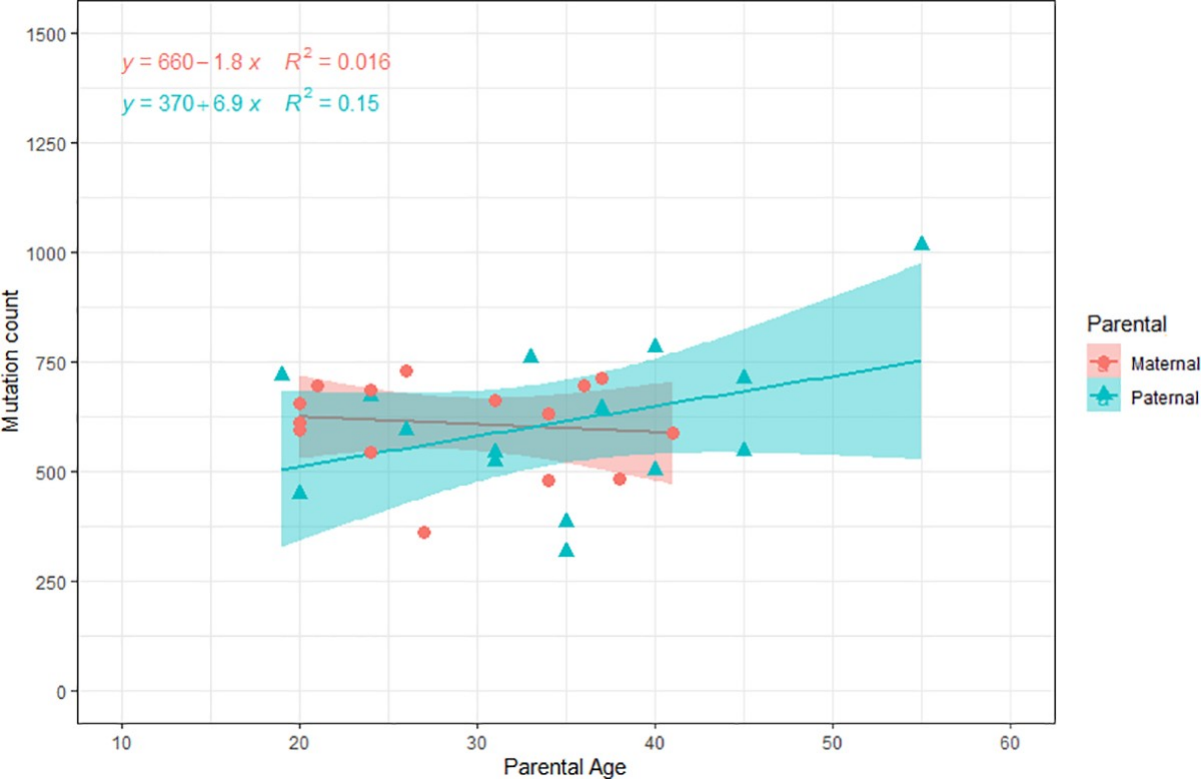
Representation maternal age effect on the number of Mendelian deviations.

With respect to the potential parental age effect, our control group revealed older fathers contributed more MDs to their offspring (Fig 5A–5C), which could be modeled by the number of mitotic spermatogonia divisions as a function of age, reinforcing previous findings regarded as male-mutation bias [39, 40]. However, our study failed to detect the maternal age effect on the number of MDs (Fig 6). Although there has been increasing evidence of maternal contributions to the *de novo* point mutations in the offspring [41, 42], others have argued that females contribute less MD to their offspring based on sex differences in gametogenesis and development [43]. To date, there is an ongoing debate about the maternal and paternal contributions to the germline mutation burden in the offspring [44]. New genomic and statistic tools applied to large and diverse populational datasets will soon help bring forth a resolution for this biological conundrum. Although larger number of family trios might be needed to assess the female contribution on the germline point mutations in their offspring, our results suggested that strength of male-mutation bias could be observed even in small family cohorts.

Single base substitutions have been a common and frequent mutational event subjacent to cell divisions spontaneously that rise as a consequence of DNA replication errors or induced by environmental stressors, such as IR. Some previously published studies on the types of DNA spontaneous base substitutions indicated all possible substitutions are well represented in germline cells [45]. Such studies suggested that transition rates tend to be higher [46] than transversion rates [47]. The findings in the current study supported these observations, since a higher proportion of transitions was observed in the children from both cases and controls.

It has been generally assumed that in groups of small sample sizes, it would be very difficult to detect the maternal age effect on the burden of point mutations in the offspring. Nevertheless, in order to test the hypothesis that in our exposed cohort germline mutations in both sexes were damage-induced by exposure to low doses of IR, we stratified our set of phased *de novo* mutations in 6 classes based on parental and derived alleles (Table 4).

**Table 4:** Summary of the descriptive data of the case and control groups for the six classes of base substitution in the genome of children conceived after parental exposure to low doses of ionizing radiation and their controls.

lGroup	Class	Minimum	Maximum	Mean	SD*	Total
Control	C>A	38	137	100.93	29.058	1,514
	C>G	45	141	111.47	28.538	3,344
	C>T	238	558	435.93	92.669	2,798
	T>A	45	84	64.67	14.034	6,539
	T>C	252	635	418.93	99.427	970
	T>G	61	141	96.67	21.091	6,284
Exposed	C>A	72	209	148.27	45.325	2,224
	C>G	90	291	168.60	56.616	2,529
	C>T	376	1.166	672.67	234.248	10,090
	T>A	48	147	85.27	27.825	1279
	T>C	307	847	561.60	161.834	8,424
	T>G	75	219	132.47	42.797	1,987

*SD = Standard Deviation.

**Fig 7:**
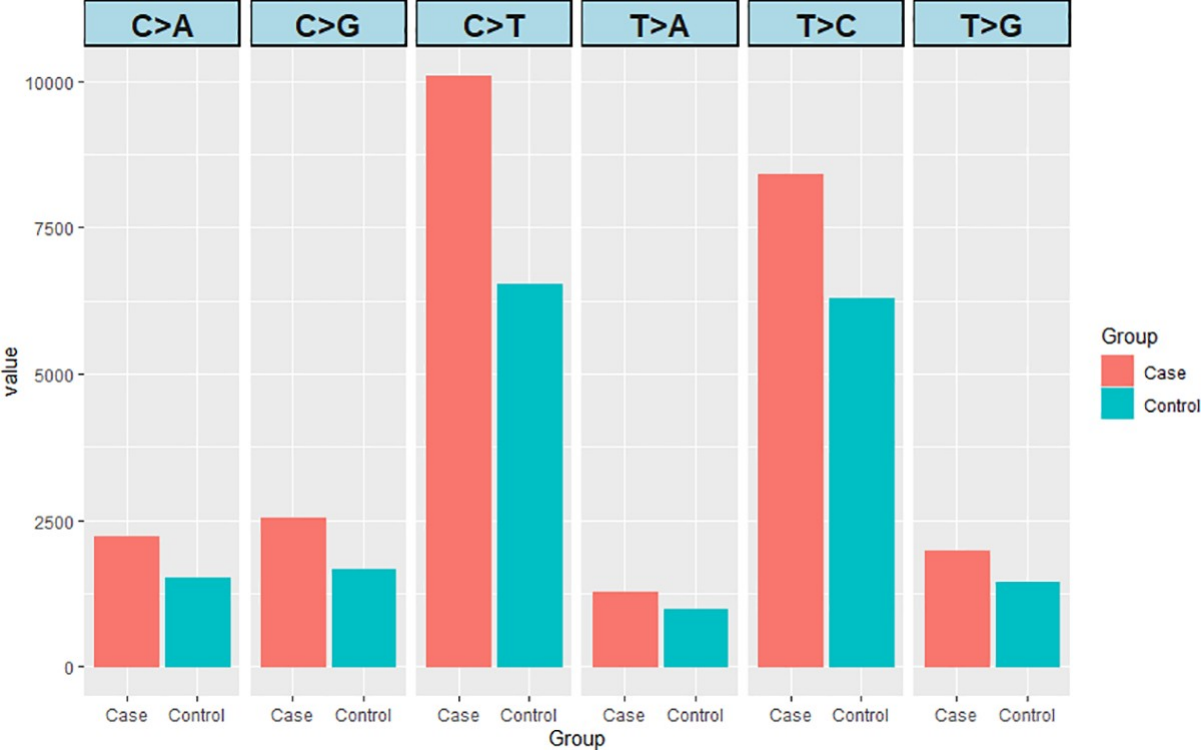
Phased de novo mutations based on parental and derived alleles distributed in classes of base substitutions for cases and controls from the Goiânia population accidentally exposed to low doses of ionizing radiation.

All the SNPs harboring C>T transitions in the data sets were not located in CpG islands and were all included in the analyses. IR is known to cause double stand brakes and all types of base substitutions. Although all transitions and transversions were observed in our data set (Fig 7), C>T and T>C were overrepresented, for both cases and controls, favoring the well-known hypothesis that human genome harbor a mutational bias toward A/T composition in the DNA stand [48]. In our study, although the base line of the MF_MD_ in SNPs were different, the mutational spectra of cases and controls, considering all base substitutions, were remarkably similar. This observation supports previous claims regarding the random effect of the deposition of radiation energy on biological systems [49].

**Fig 8:**
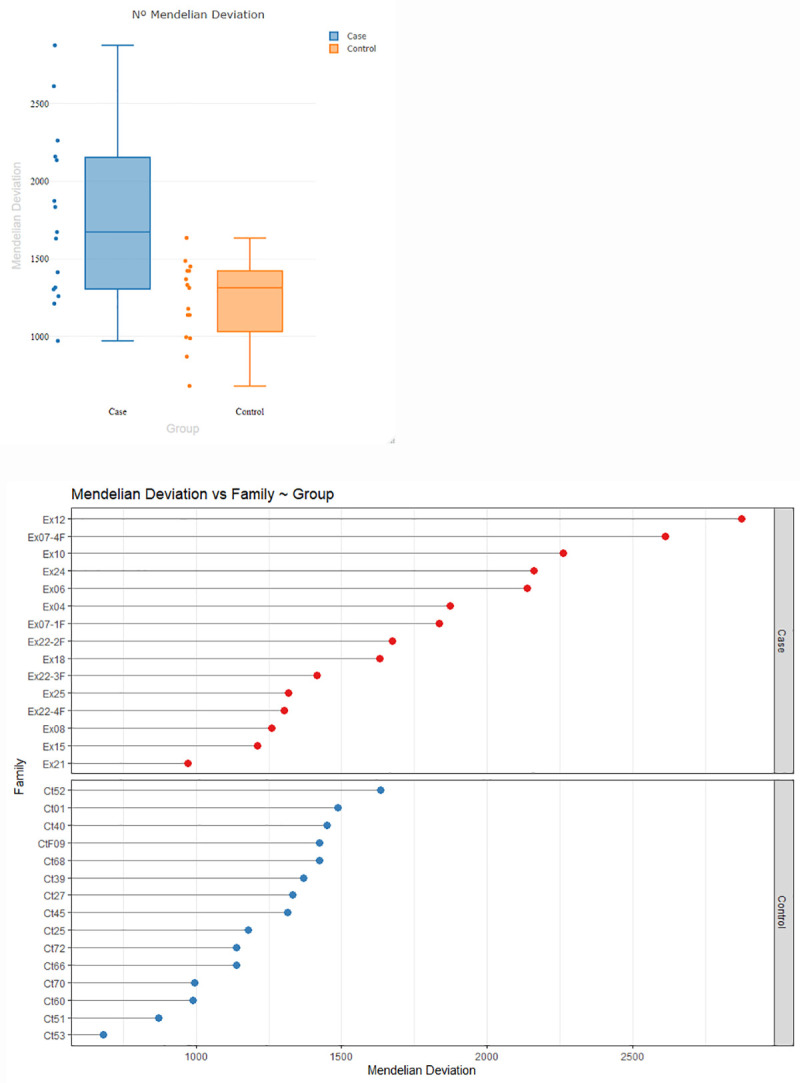
Representation of the variability of Mendelian deviations. (A) Mean numbers of Mendelian deviations in the offspring of the case and control groups. (B) Representation of the number of Mendelian deviations for each trio in the case and control groups.

In the context described before, MF_MD_ of polymorphic markers was a quantifiable and useful variable to estimate the parental contribution to the mutational burden of their children, as a consequence of transmitting non-deleterious point mutations induced by IR above the threshold expected from the control population. DNA damage in the parental germ lines could have gone uncorrected by the DNA repair system, fixed in the cells and then transmitted to the offspring. The F test, to evaluate MD frequencies in the test groups, showed the number of observed MDs were significantly different (F = 4.47; p = 8 x10^-3^). The arithmetic mean of the MD in the offspring of case and control groups are shown in Fig 8A, whereas Fig 8B shows the representation of the total of MDs observed in each family trio in both groups.

To validate the findings of the current study, which analyzed the MF_MD_ of a small cohort of children conceived after their parents were accidentally exposed to ionizing radiation from Cs-137, we suggest the application of the current study design to larger cohorts. It might be advisable to include a wider range of absorbed doses, resulting from either therapeutic or occupational exposures, to assess the potential of Mendelian deviations as retrospective biomarkers for IR exposure in human populations. In the present study, the case and control groups belonged to the same population and, therefore, were subjected to similar general environmental effects. Thus, it was safe to conclude that the average MF_MD_ was higher in the exposed group as a result of higher germline base substitutions than in the control group, which could be reasonably assumed as a consequence of parental exposure to low doses of IR. In this context, low doses of low-LET radiation induced MD in autosomal SNPs that could be identified, quantified and, therefore, used as a biomarker of effect to study human populations according to their history of exposure to environmental mutagenic insults.

## Conclusions

This study pioneered the analysis of MDs using autosomal SNP data observed in parent-offspring trios as biomarkers of effect to low doses of ionizing radiation. We succeeded estimating retrospectively the germline mutation frequency of SNPs in a human population accidentally exposed to low doses of radiation from Cs-137 and estimated the burden of germline mutations in the offspring.

We found the sex of the progenitors had no effect on the MF_MD_ of autosomal SNPs, for both case and control groups, mother and fathers contributed equal numbers of *de novo* MD to their offspring. After accounting for age, our control group revealed older fathers contributed more MD to their offspring, which could be modeled by number of mitotic spermatogonia divisions as a function of age, supporting previous findings of male-mutation bias. However, our study failed to detect the maternal age effect on the frequency of MDs.

In summary, there was a 44.0% increase in the MF_MD_ of the offspring of those accidentally exposed to low doses of IR, from a radiological accident in Goiânia. Low absorbed doses of IR could predict the increase of the mendelian deviation in the exposed group. Therefore, we concluded that MF_MD_ is a potentially useful biomarker to estimate parental exposure to IR and suitable for human population biomonitoring. In this context, future studies involving the behavior of MDs following diverse genomic and mutagenic hazards, caused by exposure to environmental agents, may provide important knowledge of the biological effects, mechanisms, and risks resulting from human exposure to such agents.

Finally, we are confident SNP array data can be used to estimate ionizing radiation-induced mutagenesis in human populations, provided the appropriate bioinformatics and statistical tools are used to extract the necessary information for biological inferences and to validate the scientific hypotheses underlying each investigation.
